# Drug Delivery Systems for Personal Healthcare by Smart Wearable Patch System

**DOI:** 10.3390/biom13060929

**Published:** 2023-06-01

**Authors:** Bikram Khadka, Byeongmoon Lee, Ki-Taek Kim

**Affiliations:** 1 Department of Biomedicine, Health & Life Convergence Sciences (BK21 Four), Biomedical and Healthcare Research Institute (BHRI), Mokpo National University, Muan-gun 58554, Jeonnam, Republic of Korea; khadkabikram180@gmail.com; 2 Soft Hybrid Materials Research Center, Korea Institute of Science and Technology, Seoul 02792, Republic of Korea; hardrist@kist.re.kr; 3 College of Pharmacy and Natural Medicine Research Institute, Mokpo National University, Muan-gun 58554, Jeonnam, Republic of Korea

**Keywords:** smart wearable patch, personalized healthcare, biosensor, drug delivery systems, stimuli-responsive carriers

## Abstract

Smart wearable patch systems that combine biosensing and therapeutic components have emerged as promising approaches for personalized healthcare and therapeutic platforms that enable self-administered, noninvasive, user-friendly, and long-acting smart drug delivery. Sensing components can continuously monitor physiological and biochemical parameters, and the monitoring signals can be transferred to various stimuli using actuators. In therapeutic components, stimuli-responsive carrier-based drug delivery systems (DDSs) provide on-demand drug delivery in a closed-loop manner. This review provides an overview of the recent advances in smart wearable patch systems, focusing on sensing components, stimuli, and therapeutic components. Additionally, this review highlights the potential of fully integrated smart wearable patch systems for personalized medicine. Furthermore, challenges associated with the clinical applications of this system and future perspectives are discussed, including issues related to drug loading and reloading, biocompatibility, accuracy of sensing and drug delivery, and largescale fabrication.

## 1. Introduction

The human skin consists of multiple layers, including the stratum corneum (SC), viable epidermis, dermis, and hypodermis [1,2]. The dermis (approximately 1 μm thick) consists of structural molecules (hyaluronic acid, elastin, and collagen), sweat glands, hair follicles, and blood capillaries and has high water content. These blood capillaries, which are enriched in the dermis and hypodermis, allow drugs applied via the transdermal route to be absorbed systemically [3]. The SC (10–20 μm thick), which is the outer barrier of the epidermis, consists of the corneocyte lipid envelop and intercellular lipid layers, which are highly interconnected [4]. The highly lipophilic and tightly connected structure of the SC impedes the delivery of molecules with high molecular weight (>500 Da) or high hydrophilicity (log P < 1) (i.e., ionic molecules) to the dermis and blood vessels [5].

The advantages of transdermal drug delivery are exhibition of controlled drug release and reduced side effects, achievement of reduced dosing schedules, ease of on/off-application, and bypassing first-pass metabolism [6,7]. However, transdermal drug delivery has so far been adopted to only limited applications. The limitation of transdermal drug delivery is attributed to the low permeability of the SC layer, insufficient drug-loading capacity, skin irritation caused by the long applying time, and low reproducibility due to the large variation in skin status among patients [7,8]. To overcome these limits, various transdermal drug delivery systems (TDDSs) have been extensively investigated and have evolved over several generations. The first generation of TDDSs, based on chemical enhancers, oil formulations, or nano-carriers, aim to passively enhance the skin penetration and diffusion of drugs. The second generation can more actively enhance those methods of skin penetration and diffusion by adjusting external stimuli such as electricity, voltage, ultrasound, and heat/light. The third generation mainly comprises microneedle formulations that can perforate the SC barrier and deliver drugs directly to the upper dermis [3].

The fourth generation, which has been actively investigated in recent years, involves thin, soft, and flexible devices that can be smoothly and tightly integrated on the skin [9]. Using these smart wearable devices, the physiological parameters (physical information such as cardiovascular signals and body temperature and the biochemical information including various biomarkers) of the user or patient can be monitored in real time, and transdermal drug delivery from the patches can be controlled by means of the various signals detected by the devices [10]. Thus, personalized therapy and healthcare can be achieved using the fourth generation of TDDSs, which are smart wearable patch systems [9,10].

This review focuses on the components of smart wearable patch systems and various types of physical and biochemical information monitored by these systems. Moreover, we discuss various drug delivery systems used in the smart wearable patches, their recent applications in specific diseases, and the limitations of further development processes, especially clinical trials.

## 2. Basic Concept of Smart Wearable Patch Systems

Smart wearable patch systems generally comprise supporting substrates, adhesive films, flexible circuits, thin-film sensor systems, actuator components, therapeutic systems, data transmission systems, power supplies, and/or energy-harvesting systems [11]. When these systems are applied on the skin, the sensor components detect and monitor various types of physical and biochemical information, such as blood pressure and blood glucose levels, from sweat. The signals from the sensors can be transferred as data, which can be viewed on a mobile device or computer in real time via a wireless data transmission system. In addition, actuator components can generate electric, thermal, and/or vibrational energy if the signals are outside the normal criteria. This form of energy transfer to therapeutic systems occurs as stimuli, and drug delivery can be triggered or enhanced by these stimuli [12]. 

**Figure 1:**
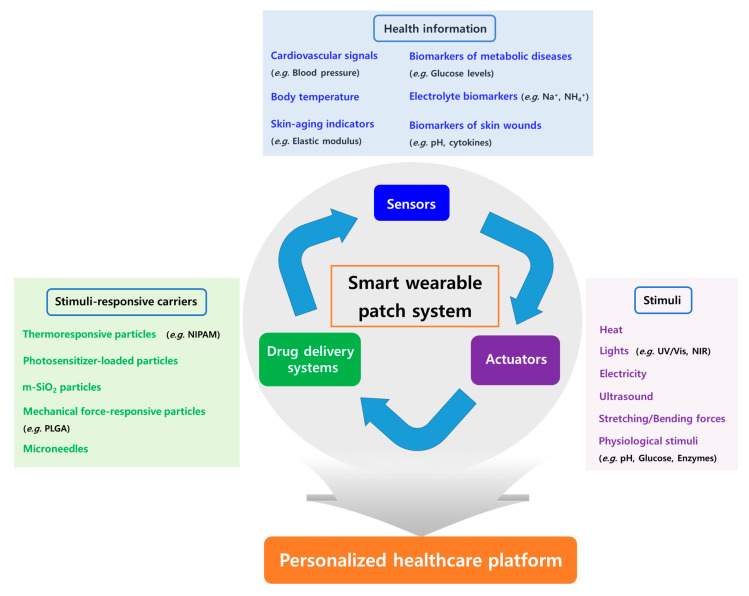
Schematic diagram of the main components of smart wearable patch system enabling per- sonalized healthcare (NIPAM: poly(N-isopropylacrylamide); m-SiO_2:_ mesoporous silica; PLGA: poly(lactic-*co*-glycolic acid); UV/Vis: ultraviolet/visible; NIR: near-infrared).

Therapeutic components can be incorporated into the substrates as the drug itself, or micro-/nano-particles can be included in other reservoirs such as hydrogels or microneedles. Drug release from these therapeutic systems to the skin and its systemic absorption can be triggered or enhanced by pathological conditions such as pH and enzymes; extrinsic stimuli such as heat, electricity, and light [ultraviolet (UV) and near-infrared (NIR)]; or mechanical movements such as stretch force [12,13,14,15]. These stimuli-responsive carrier-based DDSs that are combined with the biosensors and actuators enable one to produce closed-loop and on-demand drug delivery to users or patients, and for this reason it can be considered as a promising personalized healthcare platform (Figure 1).

The most significant characteristic of smart wearable patches is their flexibility and stretchability, which can be achieved using highly soft and stretchable polymers (e.g., polydimethylsiloxane) as a supporting substrate and fabricating essential components (e.g., sensors, electrodes, circuits, and actuators) using thin-film or miniaturization methods [16,17]. Another ideal characteristic of the wearable patches is biocompatibility, which can lead to long-term and repeated wearing without skin irritation or allergic/immune responses in the patient or user [18]. Regarding these characteristics, the soft, thin, flexible, and stretchable smart wearable patch systems that have been actively investigated possess frontier technologies compared to commercial wearable patch systems for healthcare applications (Table 1).

## 3. Physical and Biochemical Information Monitored by Sensor Components

The sensor components of smart wearable patch systems can monitor physical health information such as blood pressure, ECG, and body temperature [23]. Biosensors also detect and quantify biochemical information such as metabolites and electrolytes [24]. The continuous monitoring of health information using biosensors can be adapted for healthcare applications, disease diagnosis, and personalized therapy [10,23]. The various physical and biochemical parameters monitored by the sensor components of the smart wearable patch are summarized in Table 2.

### 3.1. Physical Information

#### 3.1.1. Cardiovascular Signals

Pressure sensors such as piezoresistive, piezocapacitive, and piezoelectric (triboelectric) sensors can monitor cardiovascular signals, including the arterial pulse and ECG, which can help manage cardiac rhythm disorders [19,25,26]. Continuous measurement of blood pressure, which is closely related to hyper-/hypo-tension and cardio-/cerebrovascular diseases, can also be achieved by those sensors that detect the arterial pulse and ECG [27,28].

Mechano-acoustic sensor components, including accelerometers, can monitor heart murmurs, which may be related to heart valve disorders, by measuring the vibration of chest skin from heartbeats [29].

#### 3.1.2. Body Temperature

Temperature-sensitive, resistance-based sensors (i.e., reduced graphene oxide (rGO) and gold (Au) as conductive materials) can detect changes in electrical resistance depending on temperature, thereby continuously monitoring body temperature. This provides information regarding overall physiological condition and skin status [30,31].

#### 3.1.3. Skin Aging Indicators

Monitoring skin humidity and UV exposure can help determine the use of cosmeceuticals, thereby delaying skin aging. Humidity sensors based on rGO or carbon nanotubes (CNTs), which have water-permeable structures, can detect changes in epidermal electrical resistance depending on the water molecules present in the skin [32]. Photosensors based on zinc oxide (ZnO), functional ZnOs, and rGO can monitor UV exposure in the skin by detecting changes in conductivity depending on the adsorption and desorption of oxygen molecules in the porous structures of these materials [33,34]. 

Continuous measurement of the elastic modulus of the skin can help attenuate wrinkles and determine areas of skin wounds and cancer. Sensor components, including piezoelectric sensors and actuators to measure the elastic modulus, can detect differences in the deformation of the elastomer layer and the skin when variable voltages are applied [35]. Generally, normal skin has a smaller elastic modulus than aged, damaged, or wounded skin areas [35]. In addition, the elastic modulus of tumor lesions in the skin differs significantly from that of normal skin [36].

### 3.2. Biochemical Information (Biomarkers) from Sweat, Interstitial Fluid (ISF), or Wounds

#### 3.2.1. Biomarkers of Metabolic Diseases

Electrochemical biosensors can quantify various metabolic biomarkers on the basis of the signals generated by specific enzyme reactions with target biomarkers from sweat or ISF [37]. Carbon-based materials (e.g., CNTs and rGO), conductive polymers (e.g., PAni), and nanowires have been widely used as materials for biosensors [23]. 

The diagnosis of diabetes through continuous glucose monitoring from sweat or ISF has been most actively studied by fabricating various types of glucose sensors based on graphene, CNT, and Au-Si/poly(amidoamine) dendrimer immobilized with glucose oxidase [38,39,40]. Monitoring lactate levels can help manage muscle conditions during anaerobic exercise and check the progress of hypoxia-derived symptoms and cardiac/liver diseases [41]. Additionally, lactate biosensors based on CNT and conductive polymers immobilized with lactate oxidase have been developed for the quantitative monitoring of lactate levels in sweat [42,43]. The detection of cholesterols and triglycerides in sweat using biosensors based on carbon materials or conductive hydrogels immobilized with enzymes (cholesterol oxide for cholesterol; lipase/glycerol kinase/glycerol-3-phosphate oxidase for triglycerides) can be utilized to diagnose hyperlipidemia and prevent cardiovascular diseases [44,45].

#### 3.2.2. Electrolyte Biomarkers

Monitoring electrolytes in sweat can be beneficial for managing body conditions during intense exercise or for specific diseases. Sodium (Na^+^), chloride (Cl^−^), and potassium (K^+^) are significant biomarkers of dehydration during exercise [46]. In particular, the concentrations of Na^+^, Cl^−^, and calcium ions (Ca^2+^) in sweat or blood are related to liver diseases such as cystic fibrosis and cirrhosis, and those of ammonium ions (NH4^+^) are related to bone diseases or wound formation [23,47,48]. Monitoring heavy metals can help manage various diseases caused by their accumulation or deficiency. Zinc (Zn) deficiency is related to immune system depletion or insulin malfunction, whereas accumulation of copper (Cu) is related to heart, kidney, and liver failure or Wilson’s disease [23]. The accumulation of cadmium (Cd), lead (Pb), and mercury (Hg) causes endocrine disruption, immunological and neurological dysfunction, and various cancers [49].

Potentiometric sensors with ion-selective membrane electrodes based on multi-walled CNTs and organic electrochemical transistors were used to identify the concentrations of Na^+^, Cl^−^, and K^+^ and Ca^2+^ and NH4^+^, respectively, in sweat during exercise [50,51]. Ion-selective membrane electrodes connected to an iontophoretic system to enhance sweat collection successfully monitored the concentrations of Na^+^ and Cl^−^ in healthy subjects and patients with cystic fibrosis [52]. Electrochemical sensors based on stripping voltammetry and Au and bismuth (Bi) electrodes identified the concentrations of heavy metals (Zn, Cu, Cd, Pb, and Hg) in sweat. This quantification was validated by analyzing urine samples using inductively coupled mass spectrometry [53].

#### 3.2.3. Biomarkers of Skin Wounds

Fluids from skin wounds contain various biomarkers related to wound formation and recovery. The pH of chronic or infected wounds can be increased to near neutral and slightly alkaline, whereas that of normal skin is slightly acidic (approximately pH 5.0) [8]. Uric acid and lactate are the major metabolites produced by impaired vascularization, hypoxic conditions, and ATP depletion in skin wounds [54]. Similarly, oxygen levels in skin wounds can be reduced. Upregulated or downregulated expression of cytokines (e.g., tumor necrosis factor α; TNF-α, transforming growth factor β; TGF-β) and interleukins (ILs) (e.g., IL-6, IL-8) have been observed in chronic wounds [55].

The pH values at skin wound sites have been identified by potentiometric sensors with positive exchange membranes and electrochemical impedance metric sensors based on CuO nanostructures [56,57]. The concentrations of uric acid and lactate in wound fluids were successfully monitored using biosensors based on porous graphene scaffolds immobilized with uricase and CNTs immobilized with lactate oxidase, respectively [58,59]. Fluorescence sensors based on phosphorescent oxygen-sensitive ruthenium compounds can visually identify oxygen levels using an external optical instrument [60]. Recently, immunosensors based on an Au nanoparticles/graphene composite immobilized with aptamers have been used to successfully monitor cytokine levels (e.g., TNF-α and TGF-β) and IL levels (e.g., IL-6 and IL-8) in skin wound fluids [61].

**Table 2:** Various physical and biochemical parameters acquired by sensor components in smart wearable patch systems.

Type of Sensor	Monitoring	Acquired Parameters	Reference
Piezocapacitive sensor	ECG	ECG	[26]
Triboelectric sensor	Arterial pulse, ECG	Blood pressure	[28]
Mechano-acoustic sensor	Vibration, ECG	Heart murmurs	[29]
Resistance-based sensor	Electric resistance	Body temperature	[30]
2D materials-based sensor	Electric resistance	Humidity	[32]
Photosensor	Conductivity	UV exposure	[34]
Piezoelectric sensor	Deformation of elastomer	Elastic modulus	[35]
Electrochemical biosensors	Conductivity	Glucose level	[38,39,40]
Electrochemical biosensors	Conductivity	Lactate level	[42,43]
Electrochemical biosensors	Conductivity	Cholesterol level	[44]
Electrochemical biosensors	Conductivity	Triglycerides level	[44,45]
Potentiometric ion sensors	Conductivity	Na^+^, Cl^−^, K^+^ levels	[50,52]
Potentiometric ion sensors	Conductivity	NH_4_^+^, Ca^2+^ levels	[51]
Electrochemical square wave anodic stripping voltammetry	Conductivity	Heavy metals	[53]
Potentiometric ion sensors/ Impedancemetric sensors	Conductivity/ Capacitance	pH value (H^+^ level)	[56,57]
Electrochemical biosensors	Conductivity	Uric acid, lactate Levels	[58,59]
Fluorescence sensor	Luminescence	Oxygen level	[60]
Electrochemical biosensors	Conductivity	Cytokines/interleukins	[61]

## 4. DDSs in Therapeutic Components

For personalized healthcare and medicine, not only smart sensor systems but smart therapeutic components must be included in the wearable patch systems. Conventional passive and diffusive DDSs (first generation of TDDSs) on the skin are always in an operational state of drug release, regardless of normal or abnormal body conditions, which limits their applications owing to side effects [9]. However, drug release from smart DDSs based on stimuli-responsive drug carriers can be activated in abnormal body conditions and suppressed when body signals are normal, enabling personalized therapy [62]. Stimuli for triggering drug release can be categorized as external stimuli (e.g., temperature, light, electrical stimulations, ultrasound, and strain) or physiological signals (e.g., pH, hypoxia, and biomolecules) [23,62]. The various stimuli-responsive carriers used in smart wearable patch systems are listed in Table 3. 

### 4.1. Stimuli-Responsive Carriers in the DDSs

#### 4.1.1. Thermoresponsive Carriers

Heat is one of the most widely used stimuli in smart DDSs and wearable patch systems. By increasing the temperature using a heat controller, thermoresponsive carriers can be degraded and dissociated, thereby triggering the release of encapsulated therapeutics [63]. Poly(N-isopropylacrylamide) (NIPAM)-based hybrid microparticles have been used as a thermoresponsive carrier [64]. Cefazolin and vancomycin as antibiotics were encapsulated in the microparticles, which were then dispersed into hydrogels loaded with vascular endothelial growth factor (VEGF) to treat skin wounds. The therapeutic systems containing these thermoresponsive drug carriers were fabricated in the form of functional threads with a heat stimulator. The release of loaded drugs increased significantly as the temperature increased, thereby enhancing the drug efficacy. Additionally, the release of cefazolin increased to a large slope at 42 °C (when the heat stimulator was turned on), whereas it was nearly stopped when the heat stimulator was turned off (at 32 °C; intact skin temperature). Thus, controlled and remote on/off drug release can be achieved using NIPAM-based wearable systems. Moreover, microneedles coated with a phase-change material (PCM) have been used as a thermoresponsive carrier [65]. Polyvinyl pyrrolidone-based microneedles containing metformin as an anti-diabetic agent were coated with tridecanoic acid as the PCM and layered with a thermal actuator. When the temperature is above the transition temperature of the PCM (approximately 42 °C), the coated PCM melts, and the loaded drug is released from the dissolving microneedles. In a diabetic mouse model, application of the PCM-coated microneedles resulted in enhanced drug release and a significant reduction in blood glucose levels when the thermal actuator was turned on. 

#### 4.1.2. Light-Responsive Carriers

Light (NIR, visible, and UV) is also a widely used stimulus in smart DDSs because of its photothermal effect. This effect causes the melting of drug carriers, the alteration of their volume, or the electronic conversion from hydrophobic state to hydrophilic state in the carriers [62]. Polycaprolactone (PCL) microneedles containing lanthanum hexaboride (LaB_6_) nanoparticles as photosensitizers have been developed to achieve NIR-triggered drug release behavior [66]. When NIR light was applied, the emission of heat from LaB6 caused the microneedles to melt, resulting in an on-off release of the encapsulated drug. This indicated a remarkable anti-cancer effect in mouse models bearing tumors in the skin layer. NIPAM-*co*-vinyl-2-pyrrolidinone (NIPAM-VP) beads containing magnetite nanoparticles (MNPs) as photosensitizers shrank because heat from the MNPs was delivered to the beads above their lower critical solution temperature (40 °C) when visible light was applied [67]. Unique release profiles depending on visible light irradiation were successfully achieved, thereby enabling on-demand and remotely controlled TDDSs. UV- and dark-triggered on-demand drug release and encapsulation were achieved using porous ZnO nanoparticles [68]. During UV-off (the dark state), the sunscreen drug was encapsulated into the ZnO nanoparticles owing to the enhanced hydrophobic interactions between the nanoparticles and the drug. When UV light was applied, the hydrophilicity of the nanoparticles increased, triggering the rapid release of the encapsulated drug. 

#### 4.1.3. Electrically Activated Systems

Electrical stimuli, such as iontophoresis, can enhance drug release and skin penetration through charge–charge repulsion between loaded drugs and electrodes [5]. Additionally, these electrically activated systems can be easily connected to sensing components and/or thermal actuators [62]. Mesoporous silica (m-SiO_2_) nanoparticles have been used as drug carriers, which can provide large drug absorption and prevent an initial burst and undesirable drug release [69]. When iontophoresis was applied, the binding force between the encapsulated doxorubicin and the nanoparticles weakened, resulting in enhanced drug release and skin penetration by electrical repulsion. Moreover, iontophoresis-driven porous microneedles containing insulin were developed for enhanced systemic absorption of encapsulated insulin in diabetic rats, which was attributed to electrical repulsion and SC layer penetration by the microneedle itself [70].

#### 4.1.4. Mechanical Force-Responsive Carriers

Some smart wearable patch systems utilize mechanical forces such as compression, stretching/bending forces, and ultrasound waves to trigger and enhance drug release at joint positions or in skin cancer [62,71]. Doxorubicin-, ciprofloxacin-, or insulin-loaded poly(lactic-*co*-glycolic acid) (PLGA) nanoparticles were dispersed in alginate microgel depots, which were half-embedded in the elastomer layer [13]. Applying stretching/bending forces and repeating the cycle promoted the release of encapsulated drugs. Additionally, this carrier can be retrieved reversibly, thereby reducing drug release when the tensile strain disappears. PLGA microplates containing curcumin were developed for the ultrasound-triggered release of curcumin [72]. These microplate systems can be combined with smart wearable patches including ultrasonic actuators to treat cancer. Insulin-loaded PLGA nanocapsules were coated with chitosan for on-demand pulsatile insulin release by ultrasound [73]. Rapid release of encapsulated insulin occurred when ultrasound was applied, whereas the release was reduced and the insulin remained within the nanocapsules when the ultrasound was turned off. 

#### 4.1.5. Physiological Signal-Responsive Carriers

Smart drug carriers responsive to physiological signals, such as pH, glucose, hypoxia, and enzymes, can be utilized as targeted DDSs applied to the skin for specific diseases, including skin wounds, skin cancer, and metabolic diseases [62]. Silica nanoparticles with outer-coated shells have been fabricated for specific drug release in infected wounds [74]. When the pH value increases to a slightly basic level because of bacterial growth in skin wounds, silane derivatives in the outer shell of the nanoparticles dissolve, releasing an encapsulated disinfectant agent into the wounds. In the cancer microenvironment, glucose levels can be elevated, and the pH is slightly acidic due to the abnormal growth of cancer cells [75,76]. Microneedles containing alginiate-coated dextran nanoparticles loaded with glucose oxidase have been developed for glucose-responsive and pH-triggered drug release to exhibit immunotherapy against skin cancer [77]. Elevated glucose in the lesion reacts with glucose oxidase in the particles, generating gluconic acid as an acidic product. This acidic product and the acidic pH in the lesion can dissociate acid-degradable dextran nanoparticles, thereby triggering the release of encapsulated anti-PD-1 into skin cancer. Additionally, microneedles containing 2-nitroimidazole (NI)-conjugated hyaluronic acid (HA) nanovesicles loaded with glucose oxidase have been developed for glucose-responsive and hypoxia-triggered drug release in diabetes [78]. In hyperglycemia, elevated glucose reacts with glucose oxidase in the particles, resulting in oxygen depletion. This hypoxic condition can cause structural changes in the hydrophobic NI chains to hydrophilic chains, thereby dissociating the nanovesicles and promoting the release of encapsulated insulin. Abnormally elevated thrombi in the blood and adjacent tissues can cause vascular occlusion and cardiovascular and cerebrovascular diseases [79]. For the treatment of thrombotic diseases, anticoagulants, including heparin, must be repeatedly administered, although the overdose can cause bleeding or hemorrhage, leading to fatal consequences [80]. Microneedles containing thrombin-cleavable peptide–heparin conjugates have been developed as thrombin-responsive, on-demand smart drug carriers [81]. Thrombin-cleavable peptides were utilized as linkers between the heparin and HA chains. In the presence of activated thrombin, the linker peptide can be cleaved, promoting the release of conjugated heparin. The pulsatile release of heparin from the prepared microneedles was confirmed, depending on the presence or absence of activated thrombin.

**Table 3:** Various DDSs based on stimuli-responsive carriers in smart wearable systems.

Type of DDSs	Triggering Stimuli	Therapeutics	Reference
Hybrid microparticles in hydrogel	Heat	Cefazolin and vancomycin	[64]
PCM-coated microneedles	Heat	Metformin	[65]
PCL microneedles containing LaB_6_	NIR light	Doxorubicin	[66]
MNP-containing NIPAM-VP beads in hydrogel	Vis light	Dexamethasone	[67]
Porous ZnO nanoparticles	UV light	Benzophenono-3	[68]
m-SiO_2_ nanoparticles	Iontophoresis	Doxorubicin	[69]
Porous microneedle	Iontophoresis	Insulin	[70]
PLGA nanoparticles in alginate microgel depots	Stretching force	Doxorubicin, ciprofloxacin, and insulin	[13]
PLGA microplate	Ultrasound	Curcumin	[72]
Chitosan-coated PLGA nanocapsules	Ultrasound	Insulin	[73]
Silica nanoparticles coated with silane derivatives	pH	Chlorhexidine	[74]
HA Microneedle containing alginiate-coated dextran nanoparticles loaded with glucose oxidase	Glucose & pH	Anti-PD-1	[77]
Microneedle containing HA-NI nanovesicles loaded with glucose oxidase	Glucose & hypoxia	Insulin	[78]
HA microneedle containing thrombin-cleavable peptide–heparin conjugates	Thrombin	Heparin	[81]

### 4.2. Fully Integrated Smart Wearable Patch Systems

The establishment of personalized healthcare and therapy requires smart wearable patch systems that can monitor various signals and biomarkers in real time, accurately control the drug release, and provide feedback. Thus, these closed-loop, on-demand, fully integrated smart patch systems (the fourth generation of TDDSs) consist of a sensing component to monitor various parameters and determine individual health states or disease progression and a DDS component to provide personalized feedback and therapy [18]. Examples of fully integrated smart wearable patch systems, including sensing and DDS components for personalized therapy, are summarized in Table 4.

An Au/Pt-based electrochemical wearable patch with a thermoresponsive PCM-coated microneedle has been reported for the monitoring and treatment of diabetes [14]. The patch system consists of three components: a sweat-uptake component, a sensing component (glucose, humidity, pH, and temperature sensors), and a therapeutic component (heater and PCM-coated microneedle). The high glucose levels, monitored from sweat, can generate heat, which leads to the melting of the thermoresponsive microneedle, thereby releasing metformin into the bloodstream. In a diabetic mouse model treated with the wearable patch system, a significant decrease in blood glucose levels (*p* < 0.01) was confirmed compared to the ‘no patch’ group. A polyimide-based electrical wearable patch with UV-responsive hydrogels has been developed for monitoring and treating infected wounds [82]. The patch system consisted of two components: a sensing component (temperature sensor) and a therapeutic component (UV-LED and hydrogel containing UV-cleavable linker-gentamicin conjugates). The elevated temperature caused by the electrical stimulus in the skin wounds can generate UV light, which cleaves the linkage and releases gentamicin into the infected wounds. In a wound infection mini pig model treated with the wearable patch system, significant inhibition of bacterial growth (*p* < 0.05) was confirmed compared to the ‘no UV light’ group. A TiO_2_/Si nanomembrane-based electrical wearable patch with thermoresponsive m-SiO_2_ nanoparticles has been reported for the monitoring and treatment of Parkinson’s disease [83]. The patch system consists of two components: a sensing component (strain and temperature sensors) and a therapeutic component (heater and m-SiO_2_ nanoparticles). Abnormal muscle strains such as tremors are monitored by sensor and generate heat, which weakens the bonding between the drug and the nanoparticles, thereby releasing anti–Parkinson’s disease agents into the bloodstream. In pig skin, deeper penetration of rhodamine B dye was confirmed when the wearable patch system was applied compared to the ‘no heating’ group. Additionally, a conductive silk fibroin hydrogel-based electrical wearable patch with a visible light–triggered, thermoresponsive hydrogel loaded with papain and gold nanoparticles has been reported for the monitoring and treatment of epilepsy [84]. The patch system consists of two components: a sensing component (strain sensor) and a therapeutic component (visible LED and silk fibroin hydrogel loaded with papain and gold nanoparticles). The detection of epilepsy by the sensor leads to the generation of visible light, resulting in the emission of heat from the gold nanoparticles and the activation of the papain enzyme, thereby releasing encapsulated phenobarbital into the bloodstream. In an epilepsy-induced rat model, the wearable patch system provided distinctive alleviation of epileptic convulsions compared to the ‘no visible light’ group. Moreover, a self-powered, triboelectric nanogenerator-based wearable patch with an iontophoresis-activated hydrogel has been developed for the monitoring of walking motion and treatment of joint injuries [85]. The patch system consists of two components: a sensing component (strain sensor and nanogenerator) and a therapeutic component (iontophoretic electrodes and poloxamer hydrogel). The detection of walking motion in the ankle by the sensor generates the electricity, which can be used as a power source for iontophoresis, resulting in the release of an encapsulated pain relief drug into the skin. In pig skin, deeper penetration of rhodamine 6G dye was confirmed when the wearable patch system was applied compared to the ‘no nanogenerator’ group.

## 5. Conclusions and Remarks for Future Research

Smart wearable patch systems integrated with sensors and therapeutic components can enable personal healthcare and medicine by monitoring various physical and biochemical parameters in real time and exhibiting on-demand closed-loop drug release. Thus, these self-administrable, noninvasive, user-friendly, and long-acting smart wearable patch systems combining biosensors and stimuli-responsive carrier-based DDSs can lead to the advent of a new era of healthcare platforms. 

However, several aspects must be addressed before the clinical application of these patch systems. The first concern is drug loading and reloading steps in the patch systems. Despite the use of a wearable patch system, macromolecules such as protein drugs, nucleic acids, and antibodies are still not suitable for transdermal delivery because of the presence of the SC barrier. High drug loading into the patch is also challenging because of the limited patch size, which can affect patient comfort and movement. Recently, these drug loading issues may be solved using a microneedle that can perforate the SC barrier and deliver macromolecules directly to the dermis. Moreover, a 3D printing method may enable higher, efficient, and reproducible drug loading.

In addition, since TDDS requires long-term use and continuous application, drug reloading into therapeutic components or the exchange of drug layers or whole patch systems needs to be thoroughly addressed. This drug-reloading process may affect patient compliance and the cost of the patch systems. Since drug reloading into the components is not a feasible process, the exchange of drug layers or whole patch systems should be considered for their long-term use. From this perspective, 3D printing technology can lead to a manufacturing revolution. This is because the technology enables reproducible mass production, which will result in a decrease in the prices of the smart wearable patches.

Another challenge is the lack of studies focusing on the long-term toxicity, long-term stability, and accuracy of sensing and drug release/delivery. The biocompatibility of the patch systems containing various components such as sensors, supporting substrate, adhesive film, flexible circuit, actuator components, therapeutic systems, and power supply and the possibility of skin irritation, local inflammatory response, or infection at patch system–applied sites (skin or epidermal/dermal tissues) should be evaluated during long-term use. However, issues related to biocompatibility and skin-related side effects may be expected not to be significant problems because numerous materials suitable for biocompatibility have been researched so far. In the case of microneedles, they can potentially cause inflammation and immune reactions as they penetrate the SC and directly reach the dermis. However, since they have entered many clinical trials (mainly vaccine formulations), it can be said that the biocompatibility of microneedle materials has been validated. Nevertheless, further research is needed to investigate any potential side effects that may arise from the long-term application of microneedles for continuous health monitoring and treatment.

Long-term stability issues must be addressed in terms of drug stability following repeated exposure to various stimuli (i.e., temperature, electricity, and NIR/UV/vis light). The stability of the various components of patch systems with repeated movements and exposure to light must also be evaluated in terms of their performance changes and their detachment from the supporting substrate or skin. Despite extensive studies on wearable sensors, the accurate detection of various signals and biomarkers from the skin remains questionable because of lower limit of quantification (LLOQ) of the biosensors and the variability in the normal range of physiological parameters in different subjects. In addition, inaccurate sensing against normal or abnormal physiological parameters can cause failure of drug release from closed-loop DDS-based therapeutic components, resulting in therapeutic failure and unwanted side effects. However, it is expected that recently researched aptamer-based biosensors will overcome the issues related to accuracy and sensitivity of detection. The advantages of aptamers in smart biosensors compared to antibodies are their higher affinity to analytes; higher stability against changes in pH, temperature, and salt; reproducibility and accurate mass production; and ease of functionalization [86]. For these reasons, aptamer-based biosensors and smart wearable patch systems incorporating them are emerging as a promising technology for point-of-care testing (POCT), point-of-care diagnostics (POCD), and personalized healthcare platforms. 

Furthermore, smart wearable patch systems are designed for personalized medicine; hence, they have limitations in terms of largescale fabrication. However, recent developments in nano-imprinting and 3D printing techniques that can be used to fabricate sensors, circuits, actuators, and therapeutic components (such as microneedles) can lead to improvements in their largescale fabrication, performance reproducibility, and cost-effectiveness.

## Figures and Tables

**Table 1:** Examples of commercial smart wearable patch systems for healthcare applications.

Commercial Product Name	Monitoring	Disease	Reference
Savvy patch ECG sensor	Electorcardiogram (ECG)	Heart rhythm disorder	[19]
Kenzen wearable device	Heart rate, Body temperature	Heat illness	[20]
MY UV Patch	UV exposure	UV-derived diseases (i.e., photoaging)	[21]
Freestyle Libre	Glucose	Diabetes	[22]
Dexcom G6			

**Table 4:** Fully integrated smart wearable patch systems for personalized therapy.

Type of Healthcare/Diseases	Monitoring by Sensors	Type of DDSs	Triggering Stimuli	Therapeutics	Reference
Diabetes	Glucose level	PCM-coated microneedle	Heat	Metformin	[14]
Infected wounds	Temperature	PEG acrylate-based hydrogels containing UV-cleavable linker	UV light	Gentamicin	[82]
Parkinson’s disease	Muscle strain (tremor)	m-SiO_2_ nanoparticles	Heat	Anti-Parkinson’s disease agent/Rhodamine B dye	[83]
Epilepsy	Mechanical sensor	Silk fibroin hydrogel loaded with papain and gold nanoparticles	Visible light/ heat	Phenobarbital	[84]
Ankle injury	Mechanical sensor	Poloxamer hydrogel	Iontophoresis	Pain-relief drugs/ Rhodamine 6G dye	[85]

## Data Availability

Not applicable.

## Abbreviations

DDSs: drug delivery systems; SC: stratum corneum; TDDSs: transdermal drug delivery systems; UV: ultraviolet; NIR: near-infrared; NIPAM: poly(N-isopropylacrylamide); m-SiO_2:_ mesoporous silica; PLGA: poly(lactic-*co*-glycolic acid); UV/Vis: ultraviolet/visible; ECG: electrocardiogram; rGO: reduced graphene oxide; Au: gold; CNTs: carbon nanotubes; ZnO: zinc oxide; ISF: interstitial fluid; Na^+^: Sodium; Cl^−^: chloride; K^+^: potassium; Ca^2+^: calcium ions; NH4^+^: ammonium ions; Zn: zinc; Cu: copper; Cd: cadmium; Pb: lead; Hg: mercury; Bi: bismuth; TNF-α: tumor necrosis factor α; TGF-β: transforming growth factor β; ILs: interleukins; VEGF: vascular endothelial growth factor; PCM: phase-change material; PCL: polycaprolactone; LaB_6_: lanthanum hexaboride; NIPAM-VP: NIPAM-*co*-vinyl-2-pyrrolidinone; MNP: magnetite nanoparticles; NI: 2-nitroimidazole; HA: hyaluronic acid; LLOQ: lower limit of quantification; POCT: point-of-care testing; POCD: point-of-care diagnostics.
